# Milk-Sickness; Its Etiology and Treatment

**Published:** 1853-06

**Authors:** Samuel W. Thompson

**Affiliations:** Albion, Illinois


Art. II.—*Milk-Sickness; its Etiology and Treatment.* Submitted to the Trustees and Medical Faculty of the University of Louisville, Feb. 1st, 1853, for the degree of Doctor of Medicine. By Samuel W. Thompson, M. D., of Albion, Illinois.

Before proceeding to give an account of the symptoms generally or invariably accompanying and therefore constituting this disease, and of the various modes of treatment pursued by those who have written upon it, I will give a short topographical sketch of the county in which I reside, and also a list of the districts in said county, where milk-sickness prevails during the autumn, which is the only time I have ever seen a case of the disease, although it is more than probable it occasionally does occur at other periods.

*Topographical sketch of Edwards County, Illinois.*—Albion, the county seat, is situated upon the water-shed or dividing ridge, between the Big Wabash river on the East, and the Little Wabash on the West. Along the eastern edge of the county runs the Bonpas creek, which empties into the Big Wabash at the south-eastern angle, or line between Edwards and Wabash counties. The ridges to the east of the town, incline from N. W. to S. S. E., so that all the water from this part of the county is taken into the Bonpas creek, and thence to the Big Wabash. Both the above streams have extensive bottom or overflown lands, varying in width from four hundred yards to two, three, or even four miles.

There is also an extensive tract of low, flat ground, about half way between Albion and the streams above spoken of, and which from its peculiar situation, without any proper outlet for drainage, is always wet and damp. It is in the immediate vicinity of this tract and of the before mentioned streams, that we see milk-sickness when such occurs in the eastern part of the county, which, however, is comparatively rare.

The northern part of the county is, with the exception of the prairie land, more broken than any other portion ; the ridges running from N. E. to S. S. W., the two sets meeting towards their heads, and thus forming a continuation northward of the water-shed. Milk-sickness is hardly known at all in these hills, except a very rare case in the close vicinity of Sugar or Village creek, both of which arise here.

To the western and north western part of the county, the land, taking Albion as the starting point, is first gently undulating, then becoming flat as it gets further towards Village creek and the Little Wabash river.

These streams each have extensive bottom lands which are overflown to a considerable depth every freshet. These lands are terminated suddenly by high bluffs. Now from a very common, but nevertheless erroneous idea, that because *low* grounds are frequently unhealthy, therefore *high* grounds must be healthy, even when in the close vicinity of unhealthy spots, the settlers have chosen these bluffs to build upon, and we are always certain to see milk-sickness in its most severe forms amongst those dwelling in this region.

The land in the south-western part of the county is rolling, with only an occasional small creek, until we get to the Little Wabash where it is again level and subject to occasional visitations of milk-sickness.

In the south and south-east the general aspect of the county does not materially differ from the previous portion, and therefore will not need a separate description.

The general character of the soil is clay, except in the prairies and bottom lands; in the former the soil is a rich black mould for a depth varying from nine inches to several feet. In the bottoms the soil is a rich black mould much mixed with portions of half decayed vegetation, as can be plainly seen by turning up the ground, when portions of half rotten twigs, roots and leaves can be found at a depth of several feet below the surface. The timber is mostly black and white oak, hickory and sweet gum, and water and laurel oak in the bottoms.

It will be seen from the above that where milk-sickness prevails, there are either creek or river bottoms in close proximity, or else low grounds interspersed with what Dr. Drake particularly mentions as being abundant in the regions where the disease originates, viz, "slashes or wet places." Dr. Drake states in his "Memoir on Milksickness," that the disease *does not* originate in the flats and overflowed lands, but in the "Elm slashes in the heavily timbered oak lands." But this I am disposed to doubt, as cattle that range exclusively in the overflowed lands and low parts of the praries on the borders of creeks are, as far as my own observation and that of others goes, most subject to the disease; and that persons residing in such neighborhoods are likewise more subject to it than those who reside on high grounds entirely away from such districts, as will be seen by referring to the sketch given above, in which it is stated that in the vicinity of Village creek and the Little Wabash, milk-sickness is of common occurrence, and that both of these streams have bottoms and low grounds on each side that are overflowed by the summer freshets; whilst on the other hand, in the northern part of the county, where there is comparatively no low grounds, milk-sickness is almost unknown. In riding through these bottom lands in the vicinity of the Little Wabash in the night time during the autumn, the smells arising from them are most offensive and sickening; one in particular I have frequently noticed. I believe I can give the best idea of it by comparing it to the odor of half rotten cucumbers, and whenever this peculiar smell is very prevalent, we expect to have sickness, and more especially milk-sickness; not because there is any evidence that this peculiar vapour or smell has anything particularly to do with the disease in question, but because we consider it as a sure indication of decaying vegetation, and therefore as testimony in itself that along with this decaying vegetation, bad or malarious vapors arise, which as I presently shall show, is most probably the true cause of milk-sickness.

In the report of the Chairman of the Committee on Practical Medicine of the Illinois State Med. Society, for 1850, it is remarked: "Of the localities of milk-sickness or trembles our information is pretty definite. Dr. Drake limits it to the white elm slashes in oak flats. Dr. Banks, formerly of Lawrenceville, in a letter to the Committee, says, 'There never has been any amount of it in Lawrence county; still a few cases have occurred in the extreme northern portion, in the clay ridges joining Crawford county, and including, from the Wabash to the Embarrass rivers; then south of Lawrenceville, in the clay, black and white oak ridges, it has been troublesome to a limited extent, and extending in small patches only, as far as Raccoon creek which divides Lawrence from Wabash county.' The general soil of Lawrence county is stated to be chiefly gravelly sand in the ridges. The general diseases to be Intermittent and Remittent, and Congestive fevers, of various grades." I would here remark, in passing, that both the Wabash and Embarrass rivers, spoken of above, and between which milk-sickness is said chiefly to occur in Lawrence county, have each very extensively overflowed lands in this part of their course.

Dr. Drake, in his memoir referred to, says, that the most plausible and probable opinion is that the disease is caused by some peculiar vegetable that the beast eats: (and therefore that man cannot contract the disease without the agency of the beast.) He inclines to the opinion that the Rhus toxicodendron is *the plant*, and quotes several cases that seem to favor this opinion. But there is a fact which is hardly consistent with this hypothesis, viz., that there is so much diversity of opinion as to what the plant is that causes all the mischief. Some thinking it is due to Dr. Drake's favorite, some to the Eupatorium ageratoides, others to the Bignona capreolata, and others again to some of the Fungi. Now this diversity of opinion could hardly exist if either of the above named articles were the cause, for in that case it would be an easy matter to test it in such a manner as to remove all doubt; but there is another circumstance which I consider fatal to this theory ; it is this : That instances have occurred in which it was perfectly demonstrable that it could not have originated in this way, examples of which I shall presently give. But one of the arguments urged by Dr. Drake against the Bignona capreolata being the cause of the disease, viz : "That other plants known to be virulent grow in and about the same places and intermingled with the Bignona," will apply with equal force to the Rhus ; for we should certainly expect that if it were the cause, we should find the disease to originate wherever this cause existed. Yet Dr. Drake says that the disease "originates in the elm slashes," although the Rhus toxicodendron grows without distinction in all parts of our forests.

The same remark applies equally to the mineral origin of the disease, for if it is dependent upon any peculiar mineral, such mineral should produce the disease whereever found ; and where the disease is present, the same mineral, be it arsenic, cobalt, or any other, should always be present. But this is by no means the case, for in Dr. Drake's pamphlet above referred to, it is stated, "That in the pools or slashes where the milk-sickness is said to originate, no special mineral has been discovered by analysis." Again; if produced by a mineral, how is it that we have the disease in Illinois where the soil is quite different from what it is in the military district of Ohio? But can we suppose it possible for any poison either vegetable or mineral to be so extensively disseminated throughout the animal tissues, that it would produce the symptoms peculiar to this poison in any animal that may have tasted the flesh or drank of the milk, in ever so small a quantity, from a beast which may have died with the disease in question ? Or that the cow whilst giving the disease to those using her milk can herself remain intact, so long as she has the milk regularly taken from her?

Yet such is one of the chief grounds upon which the theory of its animal origin is based. But if we were to admit the possibility of such extensive dissemination we are still no nearer the true cause, for we have many undoubted instances of cattle having contracted the disease in the fall, whilst feeding in a low swampy piece of meadow where they could not have got it from any mineral contained in the water, as they were supplied with this article from the same well that the family had used all through the year and yet were never troubled with the disease until the fall. This objection lies equally against the assertion that stock ranging out in the woods or prairies contract the disease from the mineral contained in the water or deposited upon the leaves by the heavy dews, for is it possible that the noxious article could lie dormant during the greater part of the year and only be found in the low grounds along creeks and ponds in the fall? In a pamphlet published some years ago by Dr. Seaton, he states that the disease is produced by arsenic which becomes mixed with the waters of White river in the district of which he speaks; but unfortunately for this theory, there is but one compound or preparation of arsenic (arsenious acid) which is at all soluble in water, and this compound can only be obtained by the agency of heat. Yet Dr. S. seems to think it is washed down from the hills in the neighborhood of this stream, in the form of white iron pyrites; but it would be the height of folly to suppose that there could be a sufficient quantity of it washed down to contaminate, even were this compound soluble, the whole of the waters of a stream as large as White river, and its effects extend no farther. But again we have milk-sickness where there is no iron pyrites, and where arsenic was never suspected to exist.

There are many facts which seem to point to its animal origin, which it is impossible in the present state of our knowledge to controvert, but nevertheless a large proportion of them can be more philosophically and satisfactorily accounted for by the theory of malaria being the cause of the disease; and those not explained upon this hypothesis must remain still enveloped in uncertainty, at least until new facts are elicited upon the subject.

As the two theories of its animal and malarious origin are closely connected in many of the facts that are generally brought forward in support of either, I shall prefer to consider them together; and if I prove that milk-sickness can and does occur in any instance without the agency of any of the causes usually designated as "animal," then we should certainly be justified, in the absence of any better substantiated theory, in attributing it to "malaria."

The advocates of the animal etiology of the disease assert that hogs, dogs, &c., as well as human beings, have been attacked by it immediately after having eaten part of the flesh of a beast dead from the trembles, when, so far as they were aware, the animals eating such meat had been subject to no malarious influences.

To this I would reply, that such animals may have been subject to such influences unknown to those recording the facts; the disease has not therefore manifested itself until the additional depressing influences of the unwholesome food from the diseased beast, has acted like the spark to a train of gunpowder, and at once caused all the untoward symptoms; just as we know that during the prevalence of cholera, a person may be, to all appearances, quite well, until he eats something, that under ordinary circumstances might produce no bad effects, but acting upon a system already weakened by the prevalent morbid cause, cholera is at once developed. But we are asked why at this particular season is milk-sickness more manifested than other diseases, in certain districts? For the same reason that during the prevalence of cholera, all other diseases are disposed to assume its livery; [illegible]o the peculiar malarious influences that I suppose to give rise to milk-sickness being prevalent, all other diseases are disposed to assume this type. But again; it is against all we know of the symptoms of this disease, to suppose that the meat alone could immediately produce it, for in all cases, or at least nearly all, authentically recorded we find more or less of the usual preliminary, or rather premonitory symptoms present; viz: great muscular debility, or an unwillingness to make any great exertion, &c., &c. These symptoms last from a few hours to as many days, and are only signs of a depressed condition of the nervous system, and are sometimes so trivial as to be unnoticed by the patient whose attention may be first seriously excited by the great prostration which comes on, and is usually, sooner or later, succeeded by nausea, &c., which I consider partly as evidence of a farther prostration of the vital powers.

We will now examine into the assertion that milk-sickness is sometimes seen without the possibility of its being received through the agency of the beast, in the shape of beef, butter, milk, &c., &c.; and as the best proof that such is the case, I will quote the following, which has come under my own observation, and for the truth of which I can therefore vouch. I. B., living in a malarious district (close to the Village creek) was subject, some ten or fifteen years ago, to periodical attacks of milk-sickness, as were also his whole family. The time of year at which this occurred, was always in the fall; at the same time of year he generally lost a good many of his cattle from "trembles." About ten years ago he fancied that he and his family contracted the disease from using beef, butter, milk, and cheese. (He never lost any other kind of stock than his cattle). From that time until within the last two years, the whole family abstained entirely from the use of any of the articles named above, or indeed of anything belonging to cattle; yet they continued to be attacked, every fall, with milk-sickness to fully as great an extent as previously. About two years ago they again took to using beef, &c., and since that time they have scarcely had a case of the disease in the house, owing, I consider, to the fact of a wet swampy piece of gronnd in the immediate vicinity having been cleared and drained during that time; and also owing partly, no doubt, to the fact that their systems were in a more vigorous tone from the use of animal diet, and thus less likely to become deranged by miasmatic influences.

Another family living in the same neighborhood, in which beef was used all through the year, were not any more subject to the disease than the previous one, yet they lived in quite as unhealthy a locality. The following will illustrate this: U. B., living in this region, had the disease in his family every fall, owing, I suppose, to his house being situated in a flat below the common level of the farm. About three years ago, his physician, Dr. S. Toompson, advised him to move his house to a higher part of his farm in the prairie. He did so, and since that time he has not had a case of milk-sickness in his house, although his cattle continue to use the same pastures as formerly, and the family to use the beef, butter, &c., as before. I could mention many other instances to the same effect, but think it unnecessary, for it seems to me, that these point so strongly to its malarious, or at least, to some other than its animal, vegetable, or mineral origin, that it is useless to multiply examples. I would merely add that U. B. continues to lose his cattle from trembles as often as he did before he moved, for they resort to the same range. To say, as some do, that miasmatic exhalations cannot be the cause, for the reason that it occasionally shows itself as it were sporadically, and then disappears when the cattle are put in tame pastures, or their milk is no longer used, is just about as philosophical as to say remittent fever is not caused by malaria, because it will sometimes show itself once in a locality, and then leave it never again to return. It is true we cannot account for all the seemingly contradictory facts said to have been observed in reference to milk-sickness, upon the supposition of its malarious origin; but I believe that a greater number of them have been satifactorily explained on this hypothesis than can be on any other.

We should also take into consideralion the fact, that many of the phenomena said to have been noticed, and upon which some of the various theories of its origin are based, are unworthy of credence, having been observed by those who were incompetent to draw just deductions therefrom, and also that many of them have only been recorded from the memory of the narrator.

Although it is an object of interest to know the exact and true etiology of milk-sickness, yet the treatment is not so much influenced by it as might be expected; but we should nevertheless spare no pains to arrive at just conclusions upon this point, and in order to assist us in this, we must turn to the pathological conditions present. But these pathological appearances can only be arrived at by repeated and careful post-mortem examinations, and here, I am sorry to say, that our authority, up to the present time, is deficient both in quantity and quality.—Such as we have, however, seems clearly to indicate a state of inflammation of the gastro-duodenal mucous membrane, and the symptoms present during the disease, would likewise lead us to the supposition that such was the case; but we should be careful how far in our diagnosis and treatment of the disease, we trust to the morbid appearances manifested after death, as we know that a mucous membrane will sometimes receive an inflammatory tint merely from a post-mortem sanguineous congestion.

We will next proceed to the *symptoms* of milk-sickness, which are very properly divided into those which are first present but very often unnoticed by the patient, and those which the physician has chiefly to deal with. The first symptoms consist of lassitude, a sense of burning and weight at the pit of the stomach; an inability to make any exertion without feeling greatly prostrated. The bowels are usually more or less costive for several days. Sometimes these symptoms do not appear, or I should rather say, are not noticed by the patient, for I believe they are always more or less present. After this state of things has continued for a time varying from one to several days, and sometimes for several weeks, it is followed in most cases by excessive nausea and vomiting. There are also muscular tremors; hence one name of the disease. The matter ejected from the stomach contains, at first, a little bile, but this soon ceases, and the patient brings up whatever is taken into this organ, mixed with dirty looking mucus, which hangs from the patient's mouth in long strings like frogs' spawn. Sometimes it is dark green, almost blue. The sense of burning and weight at the pit of the stomach is present all the time. The pulse is usually from 90 to 100, not varying much from day to day. The renal secretion does not appear to be much affected; but one of the worst symptoms is an obstinate constipation of the bowels, which is often rebellious to every ordinary purgative.

Great thirst and continual craving after cold drinks, and an inability to retain them when given, are among the most distressing symptoms. The tongue is covered with a white fur, and trembles whenever an effort is made to protrude it. Headache is a very constant accompaniment.

In some severe cases, especially towards their close, there is enormous action of the heart. In the Cyclopedia of Practical Medicine, vol. 2, page 196, we find the following in regard to milk-sickness: "The heart beats with such violence in some cases as to excite horror in the physician and bystanders. When they lay the hand upon the patient's breast, it seems to labor convulsively as though it were clogged in its action by a superabundance of blood. The patient feels nothing that he can strictly call pain, but the sense of heat at the stomach, the oppression, palpitation of the heart, and the violent efforts to vomit, constitute an extreme degree of distress." There is usually no peritoneal tenderness.

*Treatment.* The first indication in the treatment of this disease is to allay the vomiting, in order to enable the patient to keep medicine on the stomach sufficiently long to produce their several effects; but in fulfilling this indication we should, as nearly as possible, give remedies that would at the same time reduce the existing inflammation of the gastro mucous membrane, and also to remove the obstinate constipation. Some practitioners believe that the different indications are to be fulfilled by the administration of alcoholic stimulants, and thus at the same time bring on reaction and warmth, and support the sinking powers of life. But it seems to me from the general results of this mode of treatment, and from the appearances exhibited in all the recorded autopsies, that the administration of such remedies must be based upon an erroneous view of the pathology of the disease, for their obvious effects are to increase the irritation and inflammation of the stomach, if such exist; and the symptoms that have been previously stated, viz., the burning sensation at the pit of the stomach, the inability to retain anything in this organ, the insatiable thirst without heat of surface, all tend strongly to show that this state is actually present.

In a communication upon Milk-sickness, addressed to my father by Dr. J. M. Logan, of Palestine, Ill., he quotes the following case: "October 24th, 1847, called to see P. C., supposed to have milk-sickness. Symptoms—great prostration; tremor of the muscles; violent vomiting every twenty minutes; bowels had been constipated for several days; action of the heart terrific; pulse slightly accelerated and smaller than natural; surface generally cold; no soreness or pain discoverable; great restlessness." The chief treatment in this case consisted of calomel, opium and whisky, with salts and senna to open the bowels, followed by injections for the same purpose. Towards the last, when the patient was rapidly sinking, quinine and carbonate of ammonia were given in addition. Now it seems to me that the administration of whisky was not called for in this case, when we consider the symptoms of gastric derangement that were present. It is true there was an extreme degree of nervous weakness, as was made manifest by the muscular twitching and excited action of the heart. Remedies were also imperatively called for to restore warmth to the surface, but would not these ends have been better accomplished by the exhibition of small doses of calomel and bi-carbonate of soda to correct the acidity and quiet and reduce the irritation of the stomach, while at the same time we might give some of the various preparations of opium or, better still, the aqua camphoræ, and small doses of the bi-carbonate of ammonia. We must be cautious in our exhibition of opium, so as not to increase the constipation and torpor of the lower bowel, for so long as this exists all our other remedies will be comparatively useless; whilst, on the other hand, we often find that so soon as we can produce a copious alvine evacuation, all the other symptoms appear at once to be mitigated. It is also important to excite diaphoresis; we shall thus remove the chief cause of the morbidly increased action of the heart, and along with the removal of the cause, the symptoms will likewise disappear. We shall also prevent that state of collapse which is so likely to occur when the surface is suffered to continue cold. We are somewhat restricted in our use of Dover's powder, one of the best diaphoretics we have, for there is some danger of the ipecac contained in it increasing the nausea and vomiting; we must therefore depend chiefly upon some others of this class of remedies, among which the spiritus mindereri and aqua camphoræ are the best. If the symptoms imperatively call for stimulants, I should be disposed to try the latter article, which is not unpleasant to the patient, with small but repeated doses of the bicarbonate of ammonia. Another medicine which I have never seen tried, but which from its tendency to arrest vomiting in cholera might act in the same way in milk-sickness, is Dr. Stevens' saline mixture. In attempting to allay the gastric and nervous excitability, let us not for a moment forget the all important indication of opening the bowels, as the natural outlet to the secretions; for in spite of our utmost exertions, if this is neglected, the case will most likely terminate fatally; and as I have before said, if we succeed in producing free catharsis, the patient immediately begins to improve, although previously he appeared to be in an utterly hopeless condition. In the report of the Committee on Practical Medicine of the Illinois State Medical Society, we find the following on milk-sickness supplied by the chairman, Dr. Samuel Thompson. After stating the symptoms, he says of the treatment: "The indication then appears to be to open the bowels as the outlet to the secretions of the body, increased activity of which are called for in order to remove the morbid cause, be it animal ar gaseous. The difficulty in the treatment is to find medicines which can be retained long enough to have time to act. I have long been of the opinion that calomel produces its effects upon the system, only in virtue of its being absorbed into the circulation. I therefore quickly caught at the recommendation of some writer to give calomel combined with chloride of sodium. This I have found is nearly always retained and acts promptly and effectually; if necessary cream of tartar, ad libitum, is given afterwards. The calomel and salt are best given by placing them on the tongue and washing them down with water. To correct the acidity of the stomach, and because I find it gives great relief, I give bi-carbonate of soda in doses of one drachm every three or four hours, and when the constipation is removed, I give quinine, calomel and morphia." The treatment above indicated is that which I have generally seen pursued, and in most cases it has been attended with entire success.

